# Blood Collection Tubes and Storage Temperature Should Be Evaluated when Using the Siemens ADVIA Centaur XP for Measuring 25-Hydroxyvitamin D

**DOI:** 10.1371/journal.pone.0166327

**Published:** 2016-11-10

**Authors:** Songlin Yu, Weiyan Zhou, Xinqi Cheng, Huiling Fang, Ruiping Zhang, Qian Cheng, Jianhua Han, Wei Su, Liangyu Xia, Ling Qiu

**Affiliations:** 1 Department of Clinical Laboratory, Peking Union Medical College Hospital, Chinese Academy of Medical Sciences, Beijing, 100730, China; 2 Beijing Hospital National Center for Clinical Laboratories, Ministry of Health, Beijing, 100730, China; 3 Department of Clinical Laboratory, China-Japan Friendship Hospital, Beijing, 100029, China; Medical University of Gdańsk, POLAND

## Abstract

A significant bias was found when using the Siemens ADVIA Centaur XP system for measurement of 25-hydroxyvitamin D (25OHD) with VACUETTE^®^ tubes with Serum Clot Activator and Gel. Here, we examined whether other commonly used tubes or temperatures affected 25OHD results obtained with the Siemens ADVIA Centaur XP system. Serum was collected into five types of vacuum blood collection tubes from three manufacturers, and 25OHD was analyzed using the Siemens ADVIA Centaur XP system and liquid chromatography tandem mass spectrometry (LC-MS/MS) immediately or after storage at 4°C or -80°C for 48 h. Significantly higher 25OHD values were found when using the Siemens ADVIA Centaur XP system with VACUETTE^®^ tubes with serum clot activator and gel and VACUETTE^®^ tubes with clot activator but no gel compared with VACUETTE^®^ tubes with no additives. The 25OHD values in all of these tubes were not significantly different from those obtained by LC-MS/MS. Moreover, after storage at -80°C for 48 h, the values obtained in IMPROVEVACUTER^®^ tubes with serum clot activator and gel significantly increased, with a mean bias of 74.6% compared with the values before storage, on analysis with the Siemens ADVIA Centaur XP system. VACUETTE^®^ tubes containing additives significantly affect the accuracy of 25OHD results obtained using the Siemens ADVIA Centaur XP system. Additionally, the composition of serum collected in IMPROVEVACUTER^®^ tubes was affected by freezing, resulting in different measurements when using the Siemens 25OHD assay platform.

## Introduction

Siemens systems for 25-hydroxyvitamin D (25OHD) measurement are popular worldwide; however, in previous studies [1], an unexpected large bias was found when using the Siemens ADVIA Centaur XP system to measure 25OHD. This bias was related to use of VACUETTE^®^ blood collection tubes with serum clot activator and gel [2]. The mechanism mediating this interference is unclear, and whether blood collection tubes from other manufacturers also affect 25OHD measurement by the Siemens ADVIA Centaur XP system is unknown.

Furthermore, in studies of vitamin D, serum may not be measured immediately after collection. Therefore, it is also necessary to evaluate the effects of storage temperature to confirm the accuracy of vitamin D results in stored samples. However, it is unclear whether temperature affects the results obtained using the Siemens ADVIA Centaur XP system.

Here, we investigated a number of popularly used blood collection tubes from other manufacturers and with storage temperatures to confirm whether these factors affect 25OHD detection by the Siemens ADVIA Centaur XP system (Siemens Healthcare Diagnostics [Shanghai], Walpole, NJ, USA).

## Material and Methods

### Participants, study design, and analysis

Twenty healthy volunteers were recruited, and fasting blood samples were collected by venipuncture into four types of venous blood collection tubes: 5-mL BD Vacutainer^®^ SST tubes (REF: 366566, lot #4113190; BD, Franklin Lakes, NJ, USA); IMPROVEVACUTER^®^ tubes with serum clot activator and gel (REF: U222538, lot #141014; IMPROVEMEDICAL, Guangzhou, China); VACUETTE^®^ 4-mL no-additive tubes (REF: 454001, lot #A1405144; Greiner Bio-one, Kremsmunster, Austria); and VACUETTE^®^ tubes with serum clot activator and gel (REF: 454067, lot #A130202D; Greiner Bio-one). To determine whether the clot activator or gel interfered with the Siemens system, we collected serum samples from another 16 volunteers into VACUETTE^®^ 4-mL no-additive tubes, VACUETTE^®^ tubes with serum clot activator and gel, and VACUETTE^®^ tubes with clot activator but no gel (REF: 454204, lot #D140200Y; Greiner Bio-one).

The collected blood was centrifuged within 2 h (1200 × *g*, 10 min). The first aliquot was analyzed using the Siemens ADVIA Centaur Vitamin D Total kit (lot #39566029) on the Siemens ADVIA Centaur XP system according to the manufacturer’s instructions (IFU version: 10699313, 08/2012) and using a liquid chromatography tandem mass spectrometry (LC-MS/MS) method designed in our laboratory [2] immediately. The other two aliquots were separated into 2.0-mL cryovials (Corning Incorporated, USA) and analyzed after storage for 48 h at 4°C or -80°C.

This study was performed in accordance with the Code of Ethics of the World Medical Association (Declaration of Helsinki). All participants provided written consent for the use of their samples. This study was reviewed and approved by the Ethics Committee of Peking Union Medical College Hospital.

### Statistics

Statistical analyses were performed using MedCal Statistical Software (version 13.3.3; Broekstraat, Mariakerke, Beglium). Passing-Bablok regression, Bland-Altman plots, and one-way analysis of variance (ANOVA) with Bonferroni post-hoc analysis were used to compare the 25OHD results of multiple tubes. Paired t-tests were used to compare changes in 25OHD results after storage at different temperatures.

## Results

### Evaluation of the effects of blood collection tubes

**Fig 1:**
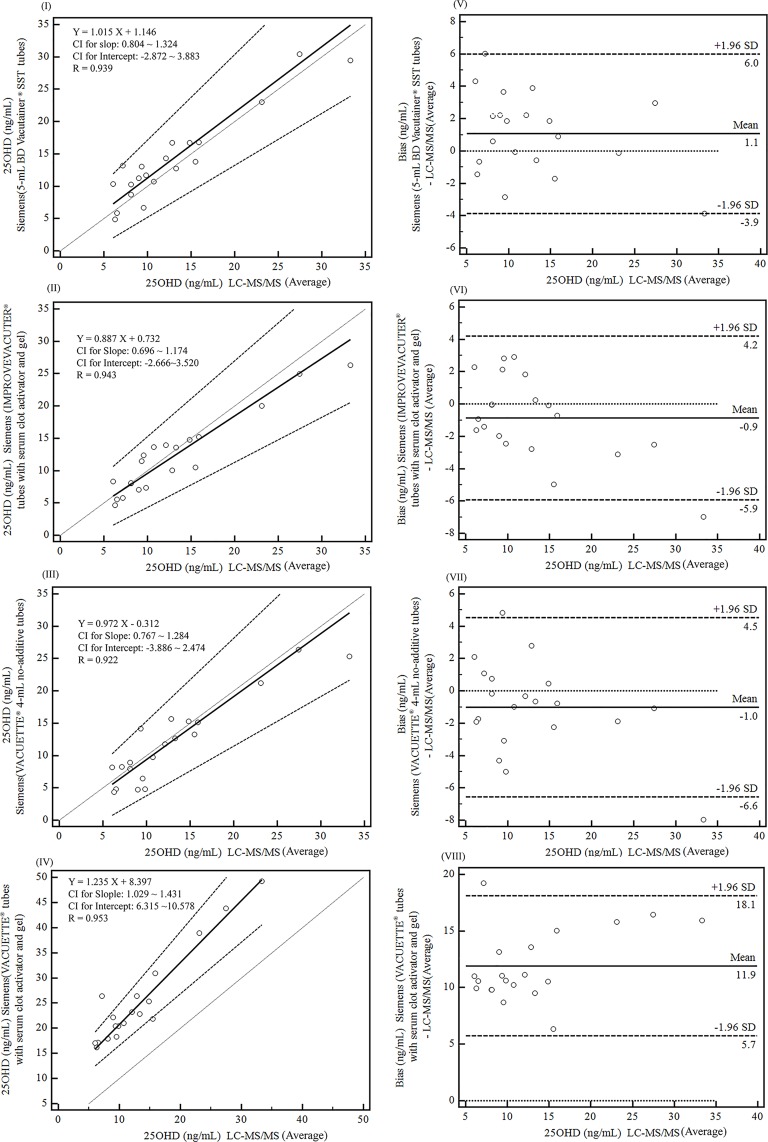
Comparison of 25OHD results obtained using the Siemens ADVIA Centaur XP system for all types of tubes and those obtained using LC-MS/MS analysis (averaged results). I–IV are Passing-Bablok regression analyses for the Siemens system (5-mL BD Vacutainer^®^ SST tubes, IMPROVEVACUTER^®^ tubes with serum clot activator and gel, VACUETTE^®^ 4-mL no-additive tubes, and VACUETTE^®^ tubes with serum clot activator and gel, respectively) and 25OHD results from the LC-MS/MS analysis (averaged results). V–VIII are Bland-Altman plots showing the 25OHD bias between the Siemens system using the four types of tubes and LC-MS/MS (averaged results).

25OHD results for LC-MS/MS were not significantly different in different tubes (*P* > 0.05). Additionally, the 25OHD results from the Siemens ADVIA Centaur XP system for all tubes correlated well with the 25OHD results of LC-MS/MS (averaged results; correlation coefficient [R] > 0.90). However, significantly higher levels of 25OHD were detected in serum collected in VACUETTE^®^ tubes with serum clot activator and gel than in serum collected using the other three types of tubes on both the Siemens ADVIA Centaur XP system (*P* < 0.01) and LC-MS/MS (*P* < 0.01). Bland-Altman tests showed that the overall biases of the 25OHD results for VACUETTE^®^ tubes with serum clot activator and gel between the Siemens ADVIA Centaur XP and LC-MS/MS were high (average: 11.9 [5.7–18.1] ng/mL; Fig 1; conversion: traditional units to SI– 1 ng/mL ≈ 2.5 nmol/L). Repeated ANOVA showed that 25OHD levels in serum collected in VACUETTE^®^ tubes with serum clot activator and gel and analyzed by the Siemens ADVIA Centaur XP system were significantly higher than those determined using LC-MS/MS (*P* < 0.001) and those determined using the Siemens system for serum collected in the other three types of tubes (*P* < 0.01; S1 Data).

In the additional samples collected from 16 volunteers, we found a similar trend for 25OHD levels in serum collected in VACUETTE^®^ tubes with serum clot activator and gel (S1 Fig, S2 Data). Additionally, 25OHD levels in serum collected in VACUETTE^®^ tubes with clot activator but no gel were 27.1% (1.2–53.0%) higher, on average, than those in serum collected in VACUETTE^®^ 4-mL no-additive tubes (*P* < 0.01; S2 Data).

### Evaluation of the effects of storage temperature

**Fig 2:**
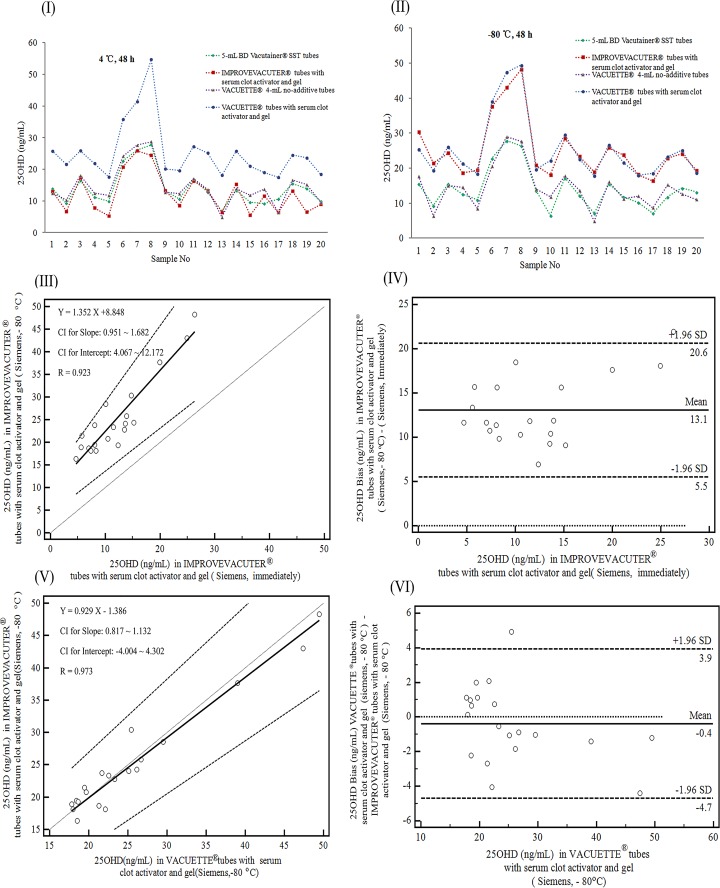
Effects of storage temperature on the measurement of 25OHD using the Siemens system. I shows the plot of 25OHD results for samples stored at 4°C for 48 h before measurement. II shows the plot of 25OHD results for samples stored at -80°C for 48 h before measurement; III and IV respectively show the Passing-Bablok regression analysis and Bland-Altman plot for 25OHD results obtained using the Siemens system (IMPROVEVACUTER^®^ tubes with serum clot activator and gel) after storage at -80°C for 48 h and immediately after collection. V and VI respectively show the Passing-Bablok regression analysis and Bland-Altman plot for 25OHD results for the Siemens system using IMPROVEVACUTER^®^ tubes with serum clot activator and gel and VACUETTE^®^ tubes with serum clot activator and gel measured after storage at -80°C for 48 h.

The 25OHD levels in all types of tubes measured using the Siemens ADVIA Centaur XP system did not change significantly after storage at 4°C for 48 h (*P* > 0.05; Fig 2I). After storage at -80°C for 48 h, the 25OHD levels in samples collected in IMPROVEVACUTER^®^ tubes with serum clot activator and gel analyzed by the Siemens ADVIA Centaur XP system were significantly increased (Fig 2II). However, the correlation between the 25OHD results in IMPROVEVACUTER^®^ tubes with serum clot activator and gel analyzed by the Siemens ADVIA Centaur XP system after storage at -80°C for 48 h and that of serum that analyzed immediately after collection was excellent (R = 0.923; Fig 2III), with a mean bias of 13.1 (5.5–20.6) ng/mL (Bland-Altman tests; *P* < 0.001 by paired *t*-tests; Fig 2IV). After storage, the 25OHD levels in serum collected in IMPROVEVACUTER^®^ tubes with serum clot activator and gel correlated well with those in serum collected in VACUETTE^®^ tubes with serum clot activator and gel (R = 0.973; bias: -0.4 [-4.7–3.9] ng/mL; *P* = 0.450; Fig 2V and 2VI) when measured using the Siemens system. Additionally, 25OHD levels in serum collected in IMPROVEVACUTER^®^ tubes with serum clot activator and gel had a significant bias compared with those in serum collected in 5-mL BD Vacutainer^®^ SST tubes (11.1 [3.9–18.3] ng/mL, *P* < 0.001) and VACUETTE^®^ 4-mL no-additive tubes (10.9 [2.9–18.9] ng/mL, *P* < 0.001) when analyzed using the Siemens ADVIA Centaur XP system after storage at -80°C for 48 h (S3 Data).

## Discussion

In this study, we compared the effects of different blood collection tubes on 25OHD results obtained using the Siemens ADVIA Centaur XP and found that when serum was collected in either VACUETTE^®^ tubes with clot activator and gel or VACUETTE^®^ tubes with clot activator but no gel, significantly higher values were obtained. Specially, in VACUETTE^®^ tubes with clot activator and gel, 25OHD on the Siemens ADVIA Centaur XP had a mean bias of 111.1% compared with that obtained by LC-MS/MS, which was significantly higher than the reference change value (RCV, 45.5%) based on a within-individual CV (CV_I_) of 12.1% [3] and the highest analytical imprecision (CV_A_, 11.1%) provided by Siemens (RCV = 2^1/2^ × Z × (CV_A_^2^ + CV_I_^2^)^1/2^, Z score of 1.96 for *p* < 0.05 [4]). Additionally, after freezing at -80°C, 25OHD in serum collected by IMPROVEVACUTER^®^ tubes showed a clinically significant mean bias of 130.1% (> RCV) compared with the results before freezing

Notably, the clot activator used by most manufacturers is micronized silica; thus, this component is common to most tubes. Additionally, the gel used in the different tubes may be all polyester and unlikely to affect vitamin D results [5]. However, the surfactants used to prevent adhesion of the cells vary, and some surfactants have the potential to interfere with immunoassays [6] because they may affect detachment of antibodies (or antigens) from solid-phase substrates [6–8]. The Siemens ADVIA Centaur assay is a competitive immunoassay using anti-FITC-labeled paramagnetic particles and a FITC-labeled vitamin D analog. Detection occurs via acridium ester (chemiluminescence), with the signal inversely proportional to the amount of vitamin D in the specimen. The label is covalently bound to the particles [9], reducing susceptibility to desorption by a surfactant, and may have led to the unexpected observation of higher 25OHD levels.

The actual 25OHD concentration itself likely did not change during the freeze-thaw process and appeared to be quite stable, even at other temperatures, as previously reported [10]. Additionally, the cryovials should not have interfered as the serum was divided into the same types of cryovials after collection, and 25OHD results in serum collected in blood collection tubes other than IMPROVEVACUTER^®^ did not show significant changes. The most probable explanation is that the composition may have changed in the IMPROVEVACUTER^®^ tubes, resulting in characteristics similar to those of the VACUETTE^®^ tubes and yielding 25OHD values that were increased to almost exactly the same degree as observed in the VACUETTE^®^ tubes with clot activator and gel. Because of the limited amount of serum obtained from the first batch of volunteers was not sufficient for application to multiple analyses, we recruited another 15 volunteers and collected serum samples in IMPROVEVACUTER^®^ tubes. These samples were then separated into three cryovials and analyzed immediately or after storage at 4°C or -80°C for 48 h by LC-MS/MS. However, no significant differences were found among the results (S4 Data).

One limitation of our study was the small sample size; however, based on a power and sample size calculation [11], for a power of 0.95, the calculated sample size was 5. Hence, the sample size used in this study was sufficient to determine the effects of tubes and temperature on 25OHD results obtained using the Siemens ADVIA Centaur XP. Another limitation was that the specific components affecting the results of 25OHD measurement on the Siemens platform were unclear because of the confidentiality maintained by tube manufacturers and Siemens. However, our results strongly suggested that VACUETTE^®^ tubes with additives should not be used on the Siemens system when measuring 25OHD. Additionally, when freezing is included before analysis, IMPROVEVACUTER^®^ tubes should also be avoided.

In conclusion, our results showed that VACUETTE^®^ tubes with additives affected 25OHD results using the Siemens ADVIA Centaur XP system. Moreover, when serum is collected with IMPROVEVACUTER^®^ tubes, freezing will affect the results obtained using the Siemens 25OHD assay platform.

## Supporting Information

S1 Data Original data for comparison of 25OHD results obtained using the Siemens ADVIA Centaur XP system for all types of tubes and those obtained using LC-MS/MS analysis. (XLS)

S2 Data Original data for evaluation of the effects of clot activator and gel on 25OHD using the Siemens ADVIA Centaur XP system. (XLS)

S3 Data Original data for evaluation of the effects of storage temperature on the measurement of 25OHD using the Siemens system. (XLS)

S4 Data Original data for evaluation of the effects of storage temperature on the measurement of 25OHD using the LC-MS/MS system. (XLS)

S1 Fig Evaluation of the effects of clot activator and gel on 25OHD using the Siemens ADVIA Centaur XP system. (TIF)
