# Supplementary figures and images for: Blood Collection Tubes and Storage Temperature Should Be Evaluated when Using the Siemens ADVIA Centaur XP for Measuring 25-Hydroxyvitamin D
Source: PLoS One. 2016 Nov 10;11(11):e0166327. doi: 10.1371/journal.pone.0166327 (PMC5104342; doi:10.1371/journal.pone.0166327)

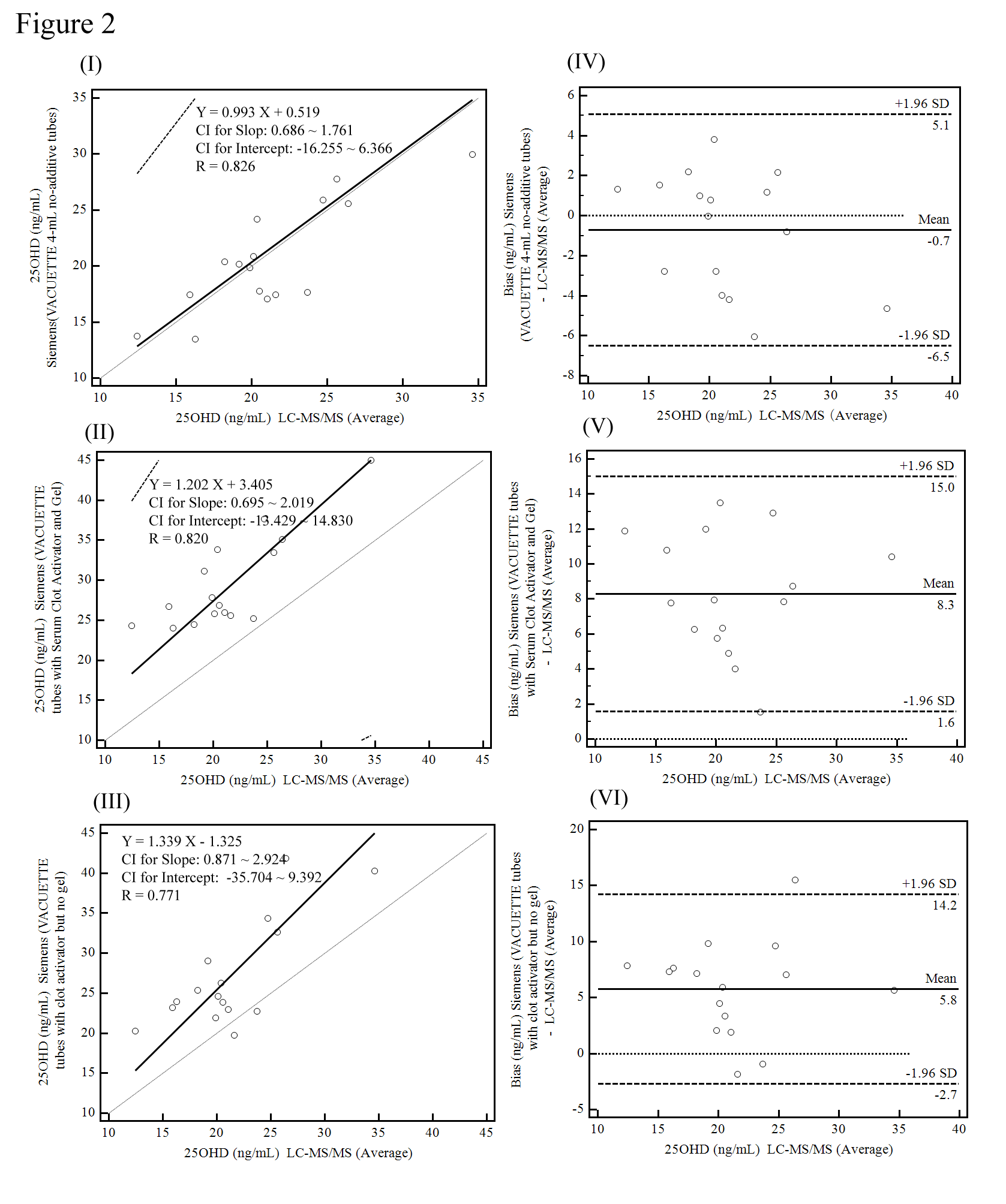

Supplement: S1 Fig — (TIF) [file pone.0166327.s005.tif]
